# Performance of Multiplex PCR and β-1,3-D-Glucan Testing for the Diagnosis of Candidemia

**DOI:** 10.3390/jof8090972

**Published:** 2022-09-17

**Authors:** Özlem Koc, Harald H. Kessler, Martin Hoenigl, Johannes Wagener, Sebastian Suerbaum, Sören Schubert, Karl Dichtl

**Affiliations:** 1 Max von Pettenkofer-Institut für Hygiene und Medizinische Mikrobiologie, Medizinische Fakultät, LMU München, 80336 Munich, Germany; 2 Diagnostic and Research Institute of Hygiene, Microbiology and Environmental Medicine, Medical University of Graz, 8010 Graz, Austria; 3 Division of Infectious Diseases, Department of Internal Medicine, Medical University of Graz, 8010 Graz, Austria; 4 Microbiology Department, St. James’s Hospital, D08 RX0X Dublin, Ireland; 5 Department of Clinical Microbiology, School of Medicine, Trinity College Dublin, The University of Dublin, St. James’s Hospital Campus, D08 RX0X Dublin, Ireland

**Keywords:** candidemia, bloodstream infection, *Candida*, multiplex PCR, β-1,3-D-glucan, antigen, serology

## Abstract

Bloodstream infections caused by *Candida* yeasts (candidemia) are associated with high morbidity and mortality. Diagnosis remains challenging, with the current gold standard—isolation from blood culture (BC)—being limited by low sensitivity and long turnaround time. This study evaluated the performance of two nonculture methods: PCR and β-1,3-D-glucan (BDG) testing. The sera of 103 patients with BC-proven candidemia and of 46 controls were analyzed with the Fungiplex Candida Real-Time PCR and the Wako β-Glucan Test. The BDG assay demonstrated higher sensitivity than the multiplex PCR (58% vs. 33%). This was particularly evident in ICU patients (60% vs. 28%) and in *C.* *albicans* candidemia (57% vs. 37%). The earlier prior to BC sampling the sera were obtained, the more the PCR sensitivity decreased (46% to 18% in the periods of 0–2 and 3–5 days before BC, respectively), while BDG testing was independent of the sampling date. No positive PCR results were obtained in sera sampled more than five days before BC. Specificities were 89% for BDG and 93% for PCR testing. In conclusion, BDG testing demonstrated several advantages over PCR testing for the diagnosis of candidemia, including higher sensitivity and earlier diagnosis. However, BC remains essential, as BDG does not allow for species differentiation.

## 1. Introduction

Yeasts of the genus *Candida* are frequently isolated in the microbiology laboratory, representing a common component of the mucosal flora of the gastrointestinal, respiratory, and female genital tracts. However, as an opportunistic pathogen, this fungus can also cause a variety of infections ranging from superficial and local infections, such as thrush or vulvovaginitis, to invasive systemic infections, including *Candida* bloodstream infection (BSI). Particularly, the most vulnerable groups, such as intensive care patients, critically ill COVID-19 patients, or hemato-oncological patients, are at specific risk of this manifestation of candidiasis [1,2,3,4]. The incidence of *Candida* BSI, which typically occurs as a nosocomial infection, has increased over time and was previously estimated to be as high as 50/100,000 hospital admissions [1,5]. Even higher candidemia rates have been reported since the beginning of the SARS-CoV-2 pandemic, which can be attributed to the novel disease entity of COVID-19-associated candidemia (CAC) [4]. *Candida* BSI is characterized by high mortality rates of up to 40% despite targeted antifungal therapy and causes more than 50,000 deaths every year worldwide [3,5,6]. The outcome has hardly improved over the last decades despite the availability of new antifungal agents such as echinocandins [1,7].

Late initiation of targeted therapy, which is a consequence of late diagnosis, is considered a major cause of the high mortality within patients suffering from *Candida* BSI [2,6]. This is due to an imperfect diagnostic gold standard, i.e., culture, which is associated with a sensitivity of as low as 50% for diagnosing invasive candidiasis [1,2,7,8,9,10]. Even if cultivation is successful, this result often comes too late due to the slow fungal growth in blood culture (BC) with a time result of several days [7,11].

In contrast, the use of nonculture methods promises more rapid results. The use of combined antigen/antibody testing is well-established in Europe and recommended by current guidelines [1,8,10]. However, to date, none of the available assays has U.S. Food and Drug Administration (FDA) clearance [12]. β-1,3-D-glucan (BDG), a fungal cell wall polysaccharide, is the target of another type of serologic assay that is not based on specific antibodies but the activation of an enzyme cascade [13]. U.S. and European guidelines recommend testing for BDG in the setting of candidemia [1,8,10,12]. Considerably, serologic assays do not allow discrimination between different *Candida* species, which is a major drawback regarding therapy management. Contrarily, molecular tests such as PCR are capable of overcoming this limitation. However, despite the increasing impact and nearly universal availability of PCR tests, the value of this technique for the diagnosis of invasive candidiasis remains uncertain. Current guidelines recognize the potential of molecular diagnostics but are concerned with the lack of comparable data and assay standardization [1,8,10,12].

This study aimed to evaluate and compare the sensitivities and specificities of a commercially available multiplex PCR and a BDG assay for the diagnosis of candidemia.

## 2. Materials and Methods

In this study, 103 sera derived from the routine diagnostics of 103 patients with BC-positive candidemia that were treated at the hospital of Ludwig Maximilian University of Munich (Munich, Germany) were analyzed. According to the EORTC/MSG criteria, candidemia was defined by the cultivation of *Candida* species from BC [9]. Of the 103 samples, 69 were also included in a previously published study, in which BDG testing and mannan antigen/anti-mannan-IgG based serology were compared [14]. The mean age of candidemia patients was 57 years and the women:men ratio was 0.58 in the case cohort. The corresponding data in the negative control cohort were 52 and 0.64. The clinical and microbiology characteristics are summarized in Table 1. Sera were obtained with a maximum interval of seven days before the date of sampling of the corresponding positive BC (day 0). Only one episode of candidemia per patient was included in the study. The sera of 46 patients with negative BC or bacterial BSI and without evidence of *Candida* BSI were included as negative controls.

All analyses were performed at the Max von Pettenkofer Institute (Munich, Germany). BC diagnostics was based on the BD BACTEC BC system (BD, Franklin Lakes, NJ, USA). Species were identified using a MALDI-TOF MS system (Bruker Daltonics, Bremen, Germany).

For molecular diagnostics, DNA extraction was performed from 1 mL of serum using a MagNA Pure Compact Nucleic Acid Isolation Kit I—Large Volume on the MagNA Pure Compact extraction system (Roche, Rotkreuz, Switzerland). An ABI 7500 FAST cycler was used for real-time PCR (Thermo Fisher Scientific, Waltham, USA). Testing for *Candida* DNA was performed with a CE/IVD-certified Fungiplex Candida Real-Time PCR kit (Bruker Daltonik, Bremen, Germany). This kit included three PCRs for the detection of *Candida* from blood samples: one *Candida* species PCR (Csp), one specific PCR for the detection of *Candida glabrata* (Cgl), and one specific PCR for the detection of *Candida krusei* (Ckr). Additionally, an extraction control was included. PCR-positive samples of the negative control cohort were reanalyzed for fungal DNA using a Bruker Fungiplex Universal Realtime PCR kit (research use only), which can detect (but not differentiate) >20 fungal genera, including *Candida*. Forty-five cycles were run for each PCR assay. Antigen testing from serum was conducted using a Wako β-Glucan Test with a cut-off of 7 pg/mL (FUJIFILM Wako Chemicals Europe, Neuss, Germany). All assays were performed according to the manufacturer’s instructions.

Significances were calculated using Fisher’s exact test (two-tailed) and McNemar’s test (two-tailed) provided by the GraphPad QuickCalcs online tool collection (GraphPad Software, San Diego, CA, USA).

## 3. Results

### 3.1. Performance of PCR Testing

**Figure 1:**
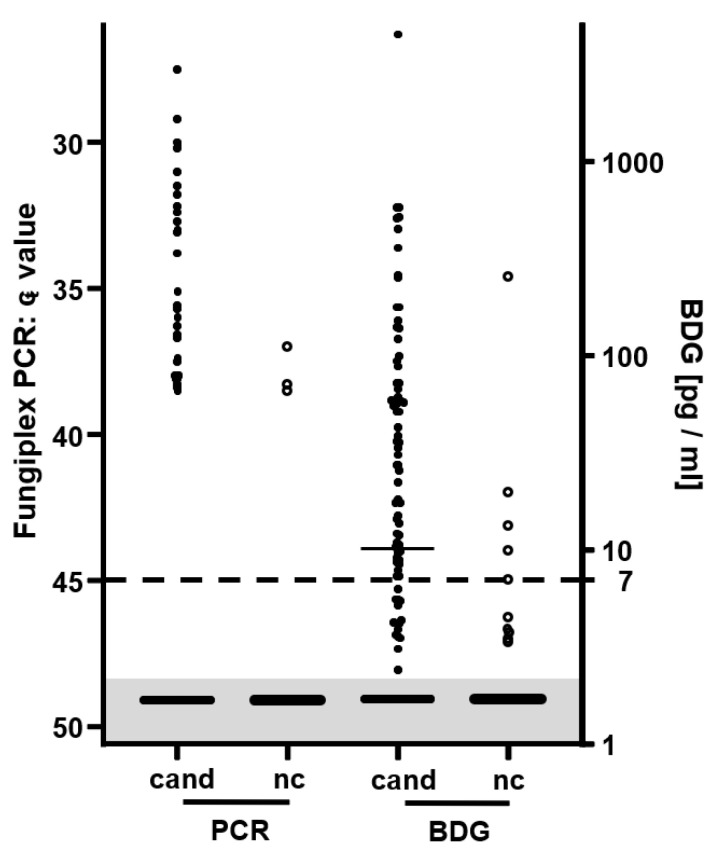
Plot of analytical measuring results of sera of candidemia patients (cand; full circles) and negative controls (nc; empty circles). The dotted line indicates the cut-off of both assays. For samples, which were tested positive in more than one PCR, only one result was considered for this graph. BDG concentrations below the limit of detection and negative PCR results are plotted (not to scale) in the grey shaded area.

With the Fungiplex Candida PCR, 34 of 103 (33%) patients with BC-proven candidemia were found to be positive, with c_t_ values ranging from 27.5 to 40.7 (Figure 1). In 31 of these 34 patients, the Csp PCR tested positive. The Cgl and Ckr PCRs identified two (c_t_ value of 32.2 and 33.1) and one (c_t_ value of 35.1) of the cases of candidemia that were missed by the Csp PCR, respectively. Another two *C. glabrata* and one *C. krusei* BSI tested positive by the Csp PCR and the specific PCRs. However, one of the *C. glabrata* positive samples was from a patient, whose BCs only grew *C. albicans.*

*Candida* DNA was significantly more likely to be detected in sera obtained closer to the sampling date of the subsequently positive BC (Table 1): sensitivity was 46% in the sera sampled in the three-day period of 0–2 days before BC and 18% in the sera sampled in the three-day period of 3–5 days before BC (*p* = 0.01). No *Candida* DNA was detected in the sera sampled 6–7 days before BC. Regarding the underlying conditions, the sensitivity ranged from 28% in a mixed ICU population to 41% in hemato-oncologic patients (not significant). For *C. glabrata* candidemia, positive results were obtained in 3/19 cases (16%). The corresponding data for *C. krusei* candidemia were 2/8 cases (25%). For the most common pathogen, i.e., *C. albicans* (52 cases), the sensitivity was determined to be 37%.

Of the 46 samples of the negative control cohort, 43 tested PCR-negative (93% specificity; Table 1). We found that 1 of 13 *S. aureus* and 2 of 15 *E. coli* bloodstream infections yielded positive test results with c_t_ values of 37.0 and 38.3 in the Csp PCR and 38.5 in the Cgl PCR. In total, 5/149 samples tested positive with the Cgl PCR. Of these samples, three were obtained from patients BC positive for *C. glabrata*, one from a patient BC positive for *C. albicans* and one from the negative control. All three PCR-positive sera of the negative control cohort were reanalyzed with a Fungiplex Universal kit: two tested negative, and one Csp PCR-positive (c_t_ value of 38.3) sample was found to contain fungal DNA (c_t_ value of 39.6).

### 3.2. Performance of BDG Testing

Of the 104 samples of patients with BC-proven candidemia, 61 tested positive with the BDG assay, with a broad range of antigen concentrations (7–4495 pg/mL; 59%; Figure 1). The highest concentrations were obtained from *C. albicans* and *C. tropicalis* BSI samples. A total of 15 sera demonstrated BDG concentrations under the cut-off and 28 sera did not contain BDG at all. BDG sensitivity was independent of sampling date, underlying clinical conditions, and fungal species (Table 1).

Of the 46 negative control cohort samples, 5 yielded positive BDG results (specificity of 89%). While one serum was found to contain >250 pg/mL BDG, the remaining positive sera were characterized by BDG concentrations close to the cut-off of 7 pg/mL (Figure 1). Three of the five samples were obtained from *E. coli* BSI patients and two from BC-negative patients. All negative control cohort samples positive for BDG tested negative in the PCR (and vice versa).

### 3.3. Comparison of Assays

When Fungiplex Candida PCR and the Wako β-Glucan Test were compared, PCR testing was less sensitive (33% vs. 58%; *p* < 0.001). The superiority of BDG analysis was evident in certain patient groups and almost absent in others (Table 1): while ICU patients particularly benefited from serologic testing (28% vs. 60%; *p* < 0.003), the selection of the test did not matter in the hemato-oncology subgroup (41% vs. 46%). While patients with *C. albicans* candidemia were better detected by the BDG assay (37% vs. 57%; *p* < 0.03), diagnosis of non-*albicans Candida* BSI was only, to a lesser extent, dependent on the test system (Table 1). BDG testing was found to be superior for the early diagnosis of candidemia, whereas PCR sensitivity continued to decline with increasing time from the date of BC sampling (Table 1). There were no significant differences in specificity.

## 4. Discussion

BC diagnostics of candidemia are impeded by several drawbacks including low sensitivity and long turnaround time [7,11,15]. This is a particular challenge in the management of *Candida* BSI patients because the outcome depends on early targeted therapy [16,17,18]. For years, there have been calls for non-culture-based methods to overcome these limitations, with high expectations for molecular techniques [7,11,15]. Since the first attempts at molecular candidemia diagnostics in the early 1990s [19,20,21], several in-house and a few commercial molecular tests have been developed, demonstrating sensitivities varying widely from 25% to 100% [19,20,21,22,23,24,25,26,27,28,29,30,31,32,33,34,35,36,37]. Probable explanations for these discrepant results include different sample volumes, different DNA extraction protocols, and particularly statistical effects due to the overall low case numbers. Only five studies had more than twenty cases included (maximum = 47), which all applied in-house PCRs with very heterogenous sensitivities of 25%, 40%, 59%, 72%, and 89% [24,25,29,32,35]. The lack of standardization is the major reason current guidelines do not recommend the use of PCR (or only as an adjunct to other methods) for the diagnosis of candidemia [1,2,8,10,12,38]. Only one pilot study investigating the Bruker Fungiplex assay was recently published. In that study, Fuchs et al. evaluated the performance of BC, Fungiplex Candida PCR, and another multiplex PCR (Roche SeptiFast test), which was discontinued in 2019 [37]. Compared with BC, the Fungiplex PCR demonstrated high diagnostic power with a sensitivity and specificity of 100% and 94%, respectively (SeptiFast: 60% and 96%, respectively). All additional SeptiFast-positive cases were also identified by the Fungiplex PCR. This excellent performance is in notable contrast to the findings of our study (sensitivity of 33% in 103 cases) but again relies on a low case number of only five cases according to the EORTC/MSG criteria [9]. The only other study to investigate a standardized, certified, and commercially available multiplex PCR, the RenDx Fungiplex kit, reported results similar to our findings (sensitivity of 44% and specificity of 87%). However, that study was also based on a very small number of only nine cases of candidemia [36]. The high variability in target sequences might be the reason for the low sensitivity of different *Candida*-specific PCR assays. For the Fungiplex Candida Real-Time PCR kit, no data on the target sequences are available.

Taking a more detailed look at our study data, the performance for detecting *C. glabrata* candidemia must be considered with some concern: despite the Fungiplex assay’s dual approach to detecting this species (Csp PCR and species-specific PCR), sensitivity was as low as 17%. Furthermore, the five Cgl PCR-positive results were distributed among one negative control, one *C. albicans* case, and three *C. glabrata* cases. However, of these three, two had already tested positive with the Csp PCR. Overall, the *C. glabrata* PCR was beneficial in only 1 of 19 *C. glabrata* BSI episodes but caused two false-positive results.

Over the last decade, BDG testing has gained an increasingly prominent role in the diagnosis of invasive candidiasis [7,12,39]. Meta-analyses concluded that both BDG sensitivity and specificity are about 80% in the setting of candidemia [7,15]. Contrarily, lower sensitivity but higher specificity, i.e., 59% and 90%, respectively, were demonstrated in our study. Presumably, this can be attributed to the use of the Wako β-Glucan Test, which is known to be more specific but less sensitive than the more widespread Fungitell assay [40]. Compared with other studies relying on the same test, the present results are consistent with expectations [41,42]. While multiplex PCRs are able to identify *Candida* at the species level, BDG analysis does not even allow differentiation between different fungal genera such as *Aspergillus, Pneumocystis,* and *Candida* [7,15]. Despite this disadvantage for serology, current guidelines favor the use of BDG for the diagnosis of candidemia over the use of PCR [1,2,8,9,10]. This is backed not only by our results (BDG and PCR sensitivity of 58% and 33%, respectively; *p* < 0.001), but also by those of previous studies [32,36]. Only the in-house assay (seminested block cycler PCR) investigated by Alam et al. in 2007 yielded better results for the molecular method than BDG [24]. However, in none of the studies relying on this PCR were data about the clinical specificity presented, which impairs proper assessment of this comparison [22,23,24]. In virtually all subgroups of our study, BDG testing had a higher sensitivity than PCR testing. There were particularly two conditions in which BDG testing significantly outperformed PCR testing: First, ICU patients had a greater benefit from a BDG than from a PCR testing approach (60% vs. 28% sensitivity, respectively). With some skepticism of this finding, however, one might object that ICU patients are particularly likely to be exposed to factors known to be associated with false-positive BDG results, e.g., immune globulin infusions or surgical materials such as sponges and gauze [43]. Second, BDG was found to yield positive results significantly earlier than PCR in sera obtained up to five days before BC sampling. In sera sampled before this date, PCR did not detect any *Candida* DNA (BDG: sensitivity of 42%). This is of particular interest as early diagnosis is pivotal, because delayed treatment initiation increases mortality [12]. Finally, a crucial factor should not be neglected when deciding between BDG analysis and PCR testing: serology is typically less expensive than molecular diagnostics.

With respect to the literature and our findings, one could question the value of molecular candidemia diagnostics. However, new techniques and noteworthy developments have been recently reported. The use of digital droplet PCR (ddPCR) promises higher sensitivities and specificities [44]. The T2Candida assay, a combination of nucleic acid amplification and magnetic resonance readout, allows skipping nucleic acid extraction, thereby reducing turnaround time. The clinical sensitivity of this novel assay was as high as 91% in a prospective multicenter study [45].

Our study significantly contributes to the overall small database concerning the value of molecular testing, particularly in comparison with BDG testing, as it included the largest cohort of candidemia cases to date applied for an evaluation of PCR performance. However, the work also has certain limitations such as its retrospective design and two constraints regarding the selection of samples. First, the cases were included based on a positive BC culture. Regarding the fact that BC can be expected to detect only about 75% of candidemia episodes [46], it must be considered that there was a selection bias: the results of this study reflect PCR and BDG performance not in the setting of candidemia but the setting of culture-proven candidemia. Second, for our analysis, the positive results of BDG and PCR testing were considered equal, which may be differently handled in clinical practice. While a positive PCR specific to Candida usually leads to therapy initiation, the significance of a single positive BDG result is still a matter of debate [18,47,48]: with regard to cost efficiency and the idea of antifungal stewardship, some experts advise against basing a therapy decision on a single positive BDG measurement alone due to its low positive predictive value [18]. Third, the fact that none of the individuals in the negative control cohort was BC-positive for *Candida* could not exclude the presence of invasive fungal infection. Therefore, designating positive samples from the negative control cohort as false positives may be precipitous. Notably, the presence of fungal DNA was confirmed in one control sample by the Fungiplex Universal PCR. However, this multiplex PCR cannot prove candidemia, because it does not differentiate between a range of different fungal species. This also applies to the BDG assay, which had a specificity of only 89%. Because no data are available concerning other invasive mycoses, e.g., *Pneumocystis* pneumonia or invasive aspergillosis, one could argue that the so-called false-positive results might just indicate another fungal infection. However, designating BDG-positive samples from the case group as true positive due to fungal infection might also be precipitous, because there are different conditions causing false-positive BDG results, e.g., bacterial coinfections, which were not excluded in our study [49]. There are also hints for BDG indicating critical illness rather than fungal infection, which presumably relies on the translocation of BDG from the gut to the bloodstream due to disrupted epithelial integrity [50]. Therefore, one could speculate that the observed early onset (several days before sampling of positive BCs) BDGemia in our patients might not have been a symptom of present candidemia but a symptom of poor health status, which predisposes the patient to subsequent fungal infection. However, the BDG and PCR specificities observed in our control group, which also included critically ill patients, are in good agreement with those reported in previous studies [36,37,41,42].

## 5. Conclusions

Our results indicate that, depending on the local availability of the assays, both tests can be used to confirm the suspicion of candidemia. The respective specificities argue against their use as screening tests. Our study suggests that BDG analysis might offer higher sensitivity than PCR testing for the diagnosis of candidemia. Positive BDG results were obtained early in the course of infection, which might allow for a timelier diagnosis. Only PCR testing offers the opportunity to identify the causative species, which allows targeted treatment. However, further studies evaluating standardized PCR assays are necessary to determine the value of molecular methods in candidemia diagnostics.

## Figures and Tables

**Table 1:** Comparison of sensitivities (a) and specificities (b) of the Fungiplex Candida PCR and the Wako β-Glucan Test.

a Candidemia Group	All Samples n (%)	PCR pos. n (%)	BDG pos. n (%)	Significance: *p* Value
Total cases	103 (-)	34 (33)	60 (58)	**0.0002**
Underlying conditions				
Hemato-oncologic malignancy	39 (37)	16 (41)	18 (46)	0.7893
HSCT	20 (19)	7 (35)	9 (45)	0.7518
Solid organ transplantation	14 (14)	4 (29)	10 (71)	0.0771
ICU	47 (46)	13 (28)	28 (60)	**0.0023**
Sampling date of serum				
0–2 days before BC	63 (61)	29 (46)	39 (62)	0.0890
3–5 days before BC	28 (27)	5 (18)	16 (57)	**0.0026**
6–7 days before BC	12 (12)	0 (0)	5 (42)	0.0736
Species isolated				
*Candida albicans*	49 (48)	18 (37)	28 (57)	**0.0244**
*Candida glabrata*	18 (17)	3 (17)	8 (44)	0.1306
*Candida parapsilosis*	12 (12)	3 (25)	8 (67)	0.1842
*Candida krusei*	8 (8)	3 (38)	6 (75)	0.2482
Others	41 (16)	7 (44)	10 (63)	0.5050
**b** **Control Group**	**All Samples** **n (%)**	**PCR neg.** **n (%)**	**BDG neg.** **n (%)**	**Significance:** ***p* Value**
Total controls	46 (-)	43 (93)	41 (89)	0.7237
BC result				
*Staphylococcus aureus*	13 (28)	12 (92)	13 (100)	1.0000
*Escherichia coli*	15 (33)	13 (87)	12 (80)	1.0000
Sterile	18 (39)	18 (100)	16 (89)	0.4795

Two-tailed *p* values were calculated using McNemar’s test with continuity correction. Results at a significance level < 0.05 are in bold. pos., positive; neg., negative; BDG, β-1,3-D-glucan; HSCT, hematopoietic stem cell transplantation; ICU, intensive care unit; BC, blood culture.

## Data Availability

The datasets generated and analyzed during the current study are available from the corresponding authors upon reasonable request.
